# Factors Affecting Universal Access to Smart Home Technologies Among Older Adults Living Alone: A Privacy–Safety Trade-Off Perspective

**DOI:** 10.3390/healthcare14152325

**Published:** 2026-08-01

**Authors:** Bo Yuan, Norazlyn Kamal Basha, Siew Imm Ng

**Affiliations:** School of Business and Economics, Universiti Putra Malaysia (UPM), Serdang 43400, Selangor, Malaysia

**Keywords:** smart home technology, older adults living alone, privacy calculus theory, adoption intention, health status, intergenerational support

## Abstract

**Background/Objectives**: Smart home technologies can mitigate the safety risks faced by older adults living alone, yet adoption remains constrained by privacy concerns. Existing research has established that privacy apprehensions deter adoption, but the conditions under which this deterrent effect strengthens or weakens are poorly understood. Drawing on privacy calculus theory, this study examines how health status and children’s support moderate the negative relationship between privacy concerns and smart home adoption intention among solo-dwelling older adults. **Methods**: A cross-sectional survey of 326 older adults living alone in three Chinese cities was analyzed using partial least squares structural equation modeling. **Results**: Privacy concerns suppressed adoption intention (β = −0.281, *p* < 0.001) while perceived safety benefits promoted it (β = 0.358, *p* < 0.001). Both moderation hypotheses were supported: the deterrent effect of privacy concerns was weaker among participants in poorer health (interaction β = −0.091, *p* = 0.039) and among those receiving stronger children’s support (interaction β = 0.137, *p* = 0.001), with the latter reducing the negative effect by approximately two-thirds. **Conclusions**: These findings demonstrate that the privacy–safety trade-off is contingent rather than fixed and inform health-differentiated privacy design and family co-management strategies for more inclusive smart home adoption.

## 1. Introduction

The United Nations projects that by 2050, the number of people aged 65 and older will surpass 1.5 billion worldwide, with solo-dwelling households among the fastest-growing segments. In China, the number of older adults living alone was estimated at approximately 31.9 million [1], a figure that has since continued to grow as urbanization draws younger family members away from their aging parents. Living alone entails safety risks—falls, cardiac events, or cognitive decline—that may go undetected for hours without a co-resident. Smart home technology offers a partial remedy: sensor-based fall detection, activity monitoring, and automated emergency alerts can compensate for the absence of a human co-resident [1,2]. Yet adoption among older adults remains low, and survey evidence across multiple countries identifies privacy concerns (fears of continuous surveillance, unauthorized data access, and loss of autonomy) as the most frequently cited barrier [3]. This gap between technological promise and actual uptake constitutes the central problem addressed in the present study.

A broad consensus holds that privacy and trust concerns rank among the most salient obstacles to technology adoption in aging populations. Systematic reviews spanning over a decade have documented this pattern, from early syntheses on aging-in-place technologies [3,4] to more recent reviews of AI-driven monitoring systems [5,6]. The empirical picture, however, is not uniformly negative: older adults sometimes accept privacy trade-offs when they perceive tangible safety benefits, particularly under conditions of elevated health risk [7,8]. This observation creates a paradox: privacy is consistently identified as a barrier, yet its deterrent force fluctuates across conditions that remain poorly specified. Existing frameworks, largely rooted in TAM and UTAUT, have predominantly tested the direct effect of privacy concerns on adoption, implicitly treating privacy as a stable attitudinal barrier and overlooking how individual health vulnerability and informal family resources may dynamically reshape the weight assigned to competing considerations. Consequently, what remains poorly understood is when and for whom the deterrent effect strengthens or weakens. Ezeudoka and Fan [9] demonstrated that anticipated regret moderated the link between digital distrust and e-health resistance, validating the broader proposition that negative factors are shaped by contextual moderators rather than operating in isolation.

Research on the psychosocial profile of solo-dwelling older adults provides further grounding for this focus. Longitudinal evidence indicates that living alone is a significant predictor of loneliness and social isolation among older adults, conditions that heighten psychological vulnerability to perceived intrusions into one’s private domestic space [10]. At the same time, qualitative studies have documented that older adults who live alone experience pronounced fear of unattended emergencies, particularly falls, a fear that intensifies with age and functional decline [11]. These dual characteristics make solo-dwelling older adults the population for whom the privacy–safety tension is most acute: they have the greatest objective need for safety monitoring yet lack a co-resident who might mediate their technology use. Despite this, the group has received remarkably little dedicated research attention.

The present study addresses this gap by examining the conditions under which privacy concerns exert a stronger or weaker effect on smart home adoption intention among older adults living alone. We draw on privacy calculus theory, which models adoption decisions as a dynamic cost–benefit trade-off rather than a static attitude-intention mapping [12,13]. This theoretical lens is particularly suited to the present context for two reasons: it foregrounds the tension between privacy costs and safety benefits that defines the daily reality of solo-dwelling older adults, and its trade-off structure provides a natural theoretical interface for introducing moderators that shift the relative weight of costs and benefits under different individual conditions. Specifically, we model two main-effect pathways (privacy concerns as a negative predictor and perceived safety benefits as a positive predictor) and introduce two moderators that capture the physical and social dimensions of vulnerability: health status (reflecting bodily frailty and urgency of safety needs) and children’s support (reflecting intergenerational assistance and emotional reassurance). The study makes three contributions: it extends privacy calculus theory from direct-effect testing to the identification of moderation mechanisms that specify when privacy concerns lose their deterrent power; it provides dedicated empirical evidence on a population whose acute safety needs and absence of co-residential support distinguish it from the broader aging demographic; and it yields actionable design implications, grounded in differentiated health profiles and family support structures, for more inclusive smart home products and services. Ultimately, this work aims to advance universal access by ensuring that smart home technology reaches the older adults who stand to benefit from it the most.

## 2. Literature Review and Hypotheses

### 2.1. Privacy Calculus Theory and Main Effects

Privacy calculus theory provides the overarching framework for this study. The theory holds that individuals engage in a cognitive trade-off between the perceived costs of privacy loss and the anticipated benefits of compliance [12]. Dinev and Hart [13] formalized this reasoning, demonstrating that privacy risks and perceived benefits exert independent, opposing effects on willingness to transact online, a calculus that has since been validated across diverse technology contexts including AIoT smart home ecosystems [14]. In the smart home domain, privacy concerns encompass surveillance discomfort, data misuse apprehensions, and perceived erosion of personal autonomy [6,7,15]. The present study treats these three facets as an integrated construct. The benefit side is anchored in safety-related gains—fall detection, activity monitoring, and emergency alerts—that carry distinctive significance for older adults living alone, for whom such technologies represent the primary mechanism for emergency detection [7]. On the cost side, empirical evidence consistently shows that privacy apprehensions deter older adults from embracing smart home technology: surveillance perceptions lower acceptance [6], privacy barriers persist across monitoring configurations [16], and ethical unease about continuous monitoring remains a source of resistance even when care benefits are acknowledged [15]. On the benefit side, systematic reviews confirm that smart home technologies yield tangible safety outcomes that older users recognize and value [5,17], and individual-level evidence shows that perceived usefulness and safety expectations significantly predict adoption [18]. Schomakers and Ziefle [7] demonstrated that older adults explicitly weigh privacy costs against security benefits, confirming that the privacy calculus mechanism operates in this population. Taken together, the evidence supports the expectation that both sides of the calculus independently predict adoption intention:

**H1.**  *Privacy concerns are negatively associated with smart home adoption intention.*

**H2.**  *Perceived safety benefits are positively associated with smart home adoption intention.*

### 2.2. The Moderating Role of Health Status

While H1 and H2 specify the two sides of the privacy–safety calculus, they leave open a critical question: does the calculus shift when individual circumstances change? The first moderator considered is health status. Prior research suggests that the relationship between health conditions and technology acceptance among older adults is not simply additive but may reshape the weight assigned to competing considerations. Arthanat et al. [19] found that functional limitations and chronic health burdens were associated with greater openness to assistive technology, and Wilkowska et al. [8] observed that older adults with more pressing health needs placed less emphasis on privacy risks when evaluating ambient health systems. More recent evidence reinforces this pattern: a systematic scoping review of digital health acceptance for chronic disease management reported that older adults with multiple comorbidities displayed higher receptivity to monitoring technologies, driven by the urgency of their health management needs [20], and a comparative study by Shi et al. [21] demonstrated that older adults in poorer health were significantly more willing to use and pay for smart home services than their healthier counterparts. The underlying mechanism can be articulated through the lens of protection motivation theory [22]. When individuals perceive themselves as physically vulnerable, facing elevated risks of falls, cardiac events, or unmanaged chronic conditions, a protective motivation is activated that elevates the salience of threat-reducing options. In this heightened state, the anticipated safety gains of smart home technology become psychologically more urgent, and privacy costs are discounted relative to the imperative of self-protection. The expected consequence is an attenuated link between privacy concerns and adoption resistance. This reasoning parallels a broader pattern in the health technology literature. Ezeudoka and Fan [9] demonstrated that anticipated regret (the fear of regretting non-adoption) significantly attenuated the relationship between digital distrust and e-health resistance among older adults. Although the specific moderator differs, the structural logic is analogous: a contextual factor that amplifies the perceived cost of inaction diminishes the deterrent power of a negative psychological state. For older adults living alone, the moderating role of health is likely amplified further. Research on fear of falling has consistently shown that solo-dwelling older adults report significantly higher levels of fall-related anxiety than their co-residing counterparts, and that this anxiety intensifies with declining health and functional capacity [11]. Poor health combined with the absence of a co-resident thus creates a condition of double vulnerability, one in which the urgency of technological protection is reinforced by the lived experience of unattended health crises, making it psychologically difficult to override by privacy reservations alone. Accordingly, we hypothesize:

**H3.**  *Health status moderates the negative effect of privacy concerns on adoption intention, such that the effect is weaker among older adults in poorer health.*

### 2.3. The Moderating Role of Children’s Support

Beyond health status, the social resources an older adult can draw on may reshape the privacy–safety calculus, and the most consequential of these for our population is the support of adult children. This support does not operate in a cultural vacuum. Family life in China remains strongly shaped by Confucian norms of filial piety, which treat care for aging parents as an obligation rather than a matter of goodwill, and this expectation lends a child’s advice and reassurance considerable weight when an older parent weighs whether to trust an unfamiliar device. We therefore expect children’s support to carry particular force as a moderator in this setting, while acknowledging that some of that force may be culturally specific—a caveat we revisit under generalizability. Intergenerational support has been identified as an important facilitator of technology adoption in later life. Wei et al. [23] showed that positive intergenerational relationships significantly enhanced community-dwelling older adults’ willingness to use smart home technology, and Zhou et al. [24] found that family encouragement and guidance were among the strongest predictors of smart home adoption intention in a Chinese sample. These findings establish that children’s support promotes adoption, but the mechanism through which it may buffer the deterrent effect of privacy concerns requires closer examination. Children’s support operates along two complementary pathways. Instrumentally, when adult children help install, configure, and manage smart home devices and oversee the data these devices generate, the older parent’s sense of privacy vulnerability is directly mitigated: the perception that a trusted person is managing one’s data transforms privacy from an uncontrolled risk into a delegated responsibility, thereby reducing the extent to which privacy concerns suppress adoption willingness. Jin et al. [25] documented a closely related mechanism, showing that intergenerational support fostered technology-specific trust, which in turn facilitated adoption among older adults. Emotionally, encouragement, reassurance, and explicit endorsement from children function as a trust endorsement. Older adults tend to regard their children as credible judges of technological safety, and a child’s recommendation signals that the privacy risks are manageable and the benefits worth pursuing. This emotional backing does not eliminate privacy concerns but dampens their psychological weight in the adoption decision. Cui et al. [26] lent further nuance to this picture through a latent profile analysis revealing that different configurations of intergenerational support (instrumental, emotional, or combined) produced distinct patterns of digital media engagement among older adults, underscoring that the type and intensity of support matter, not merely its presence or absence. For older adults living alone, children’s support assumes a distinctive character. Because the adult child is physically absent from the household, support is necessarily delivered at a distance, whether through phone guidance, remote device management, or periodic in-person visits. This remote involvement constitutes a social compensation mechanism: it partially offsets the structural disadvantage of solo living by extending a layer of informational and emotional security that co-residing family members would otherwise provide organically. In this sense, children’s support does not merely add to the benefit side of the calculus; it actively reduces the perceived cost on the privacy side by embedding the technology within a trusted relational context. We therefore hypothesize:

**H4.**  *Children’s support moderates the negative effect of privacy concerns on adoption intention, such that the effect is weaker among older adults who receive stronger support from their children.*

### 2.4. Summary of the Research Model

**Figure 1:**
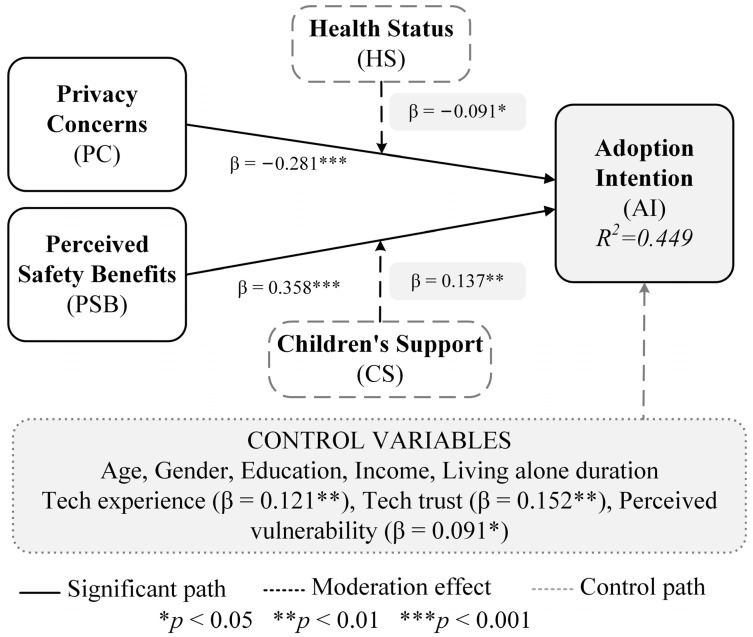
Research Model with Standardized Path Coefficients. Note. Solid lines = significant direct paths; dashed lines = moderation effects; dotted lines = control paths. * *p* < 0.05, ** *p* < 0.01, *** *p* < 0.001. Non-significant demographic controls (age, gender, education, income, living alone duration) are omitted for clarity.

In summary, the model comprises two main-effect pathways (H1–H2) and two moderation pathways (H3–H4), positing that the privacy–safety trade-off tilts in predictable ways depending on the older adult’s health profile and family support resources. The proposed research model is depicted in Figure 1.

## 3. Method

### 3.1. Sample and Data Collection

A cross-sectional survey was conducted among older adults living alone in three cities in eastern and central China. Participants were recruited through community neighborhood committees, senior universities, and community-based eldercare centers. Inclusion required being aged 60 years or older, having lived alone for at least six months, reporting no cognitive impairment, and demonstrating basic awareness of smart home technology as verified by a screening item. This screening item asked whether respondents had previously heard of home devices that can detect falls, raise emergency alerts, or monitor daily activity remotely. Before they answered, a research assistant read out a short standardized description of smart home technology so that all respondents worked from the same referent. Those who still did not recognize the concept were not enrolled. Trained research assistants administered the questionnaires primarily through face-to-face sessions with one-on-one guidance; an online version served as a supplementary channel. Data collection took place between September and November 2024. A pilot test with 30 respondents led to minor wording adjustments. A total of 430 questionnaires were distributed, of which 391 were returned (gross response rate = 90.9%). After removing cases with incomplete responses, straight-line answering patterns, or failure to satisfy the inclusion criteria, 326 valid questionnaires were retained for analysis, corresponding to an effective response rate of 75.8%. Of the 326 valid respondents, 258 (79.1%) completed the questionnaire face-to-face and 68 (20.9%) online; the three cities contributed 118 (Nanjing), 112 (Wuhan), and 96 (Hefei) respondents. A supplementary analysis confirmed that hypothesized results did not differ between administration modes. The study received ethical approval from the institutional review board of Xinxiang University, and all participants provided informed consent.

### 3.2. Measures

The survey instrument comprised 30 quantitative items (24 Likert-scale items for the latent constructs and 6 demographic items) and three open-ended questions, with an estimated completion time of no more than 20 min. All multi-item latent constructs were measured on a five-point Likert scale ranging from 1 (strongly disagree) to 5 (strongly agree). Items were drawn from established instruments in the technology acceptance and aging-in-place literatures and adapted to the specific context of smart home adoption among older adults living alone; the adaptation process involved forward–backward translation between English and Chinese, followed by review from two domain experts and the pilot test described above. Privacy concerns were captured with four items adapted from Dinev and Hart [13] and contextualized to the smart home setting, addressing worries about continuous data collection, in-home surveillance, and the risk of unauthorized access to personal information. Perceived safety benefits were assessed with four items adapted from Pal et al. [18] and Schomakers and Ziefle [7], reflecting expectations of fall detection, emergency alerts, and real-time activity monitoring that smart home devices could offer. Adoption intention was measured with three items tapping willingness and likelihood to adopt smart home technology in the near future [27,28].

Two moderating variables were included. Health status was operationalized with three items adapted from the SF-12 health survey framework (one self-rated overall health item and two items concerning chronic disease burden), with higher scores indicating better perceived health. Children’s support was measured with four items adapted from Wei et al. [23] and Jin et al. [25], capturing both instrumental assistance (e.g., helping install or manage devices) and emotional encouragement (e.g., reassurance regarding technology use) provided by adult children.

Six demographic characteristics served as control variables: age, gender, education level, monthly income, duration of living alone, and smart device experience. Two additional theory-informed controls were measured: technology trust (three items) and perceived vulnerability (three items). A recent meta-analysis has confirmed that scales grounded in the technology acceptance and protection motivation frameworks demonstrate acceptable psychometric properties when applied to older adult samples in health technology contexts [29]. Health status and perceived vulnerability are related but should not be conflated. Health status reflects how well respondents judge their body to be functioning at present, including their chronic-disease burden, whereas perceived vulnerability is a judgment about future risk—how likely and how serious a fall or an unattended emergency might be. The two can pull apart: someone may consider their current health acceptable yet still expect real danger ahead, or the reverse. Their low correlation (r = 0.231) and HTMT ratio (0.287) bore out this separation empirically, and a robustness check confirmed that every hypothesized path held with and without perceived vulnerability in the model.

### 3.3. Data Analysis

Partial least squares structural equation modeling (PLS-SEM) was performed using SmartPLS 4 (version 4.1.1.4; SmartPLS GmbH, Monheim am Rhein, Germany; https://www.smartpls.com/) [30]. PLS-SEM was selected over covariance-based SEM because the model includes product-indicator interaction terms, the data exhibited significant multivariate non-normality (Mardia’s test, *p* < 0.001), and the research objective is primarily predictive and exploratory [30]. The analysis proceeded in two stages: the measurement model was first evaluated by examining indicator reliability (outer loadings), internal consistency (composite reliability), convergent validity (average variance extracted) [31], and discriminant validity via the heterotrait–monotrait ratio of correlations (HTMT) [32]; the structural model was then assessed by inspecting path coefficients, the coefficient of determination (R^2^), predictive relevance (Q^2^), and effect sizes (f^2^). Moderating effects were estimated through the product-indicator approach, and simple slope analyses were conducted at one standard deviation above and below the mean of each moderator to probe the form of significant interactions. An importance–performance map analysis (IPMA) was additionally carried out to identify constructs that exhibit high importance for the target construct yet comparatively low performance, thereby pinpointing priority areas for practical intervention [33]. All inferential tests relied on 5000 bootstrap subsamples with bias-corrected and accelerated (BCa) confidence intervals [30]. Predictive relevance was assessed through a blindfolding procedure with an omission distance of 7. Responses to the three open-ended questions were analyzed by means of thematic coding: two researchers independently coded the transcripts and subsequently reconciled discrepancies through discussion, with inter-rater reliability assessed using Cohen’s kappa coefficient [34]. Descriptive statistics, the exploratory factor analysis for Harman’s single-factor test, Mardia’s multivariate normality test, and Cohen’s kappa were computed in IBM SPSS Statistics (version 27.0; IBM Corp., Armonk, NY, USA; https://www.ibm.com/products/spss-statistics (accessd on 1 January 2026)).

### 3.4. Common Method Bias

Because all variables were collected from the same respondents at a single point in time, several procedural and statistical remedies were implemented to mitigate common method bias [35]. Procedurally, participant anonymity was guaranteed, item order was partially randomized, and predictor and criterion variables were presented on separate pages. Statistically, Harman’s single-factor test was performed: an exploratory factor analysis with all measurement items loaded onto one factor yielded a maximum explained variance of 29.3%, well below the 50% threshold. In addition, following Kock’s [36] full collinearity-based procedure for common method bias detection in PLS-SEM, all variance inflation factors from a full collinearity assessment ranged from 1.08 to 2.21, remaining well below the conservative threshold of 3.3, indicating that common method bias is unlikely to pose a serious threat to the validity of the findings.

## 4. Results

### 4.1. Descriptive Statistics and Measurement Model

Table 1 presents the demographic profile of the 326 participants. The mean age was 71.4 years (SD = 6.2), with 62.0% of respondents being female. The largest age group was 70–74 years (27.3%), and the majority had a middle-school education (34.3%) with a monthly income between 2000 and 3999 RMB (41.1%). More than three-quarters of participants had been living alone for one to ten years (78.5%), and 85.6% reported at least one chronic disease. Regarding technology exposure, 60.7% had experience limited to basic smartphone use, 22.1% had no smart device experience at all, and only 17.2% reported some prior exposure to smart home products. Descriptive statistics for the key latent constructs are reported in Table 2 (Panel B). Mean scores on a five-point scale were as follows: adoption intention (M = 3.14, SD = 0.94), perceived safety benefits (M = 3.78, SD = 0.82), privacy concerns (M = 3.52, SD = 0.89), health status (M = 2.83, SD = 0.91), and children’s support (M = 3.41, SD = 0.96).

The measurement model was assessed following established PLS-SEM reporting guidelines [30]. As shown in Table 2 (Panel A), all indicator loadings exceeded the 0.70 threshold, with values ranging from 0.762 (HS3) to 0.912 (AI2), confirming adequate indicator reliability. Panel B of Table 2 reports composite reliability (CR), average variance extracted (AVE), and discriminant validity indices. CR values ranged from 0.852 (health status) to 0.934 (adoption intention), all surpassing the 0.70 benchmark [31]. AVE values ranged from 0.659 to 0.825, uniformly above the 0.50 threshold, thereby establishing convergent validity [31]. Discriminant validity was evaluated using two criteria. First, the square root of AVE for each construct (diagonal entries in Panel B) exceeded all corresponding inter-construct correlations in the same row and column. Second, all HTMT ratios (above the diagonal) fell below 0.90, with the highest value being 0.523 (PSB–AI), satisfying the conservative criterion recommended by Henseler et al. [32]. The common method bias tests reported in Section 3.4 further supported the integrity of the measurement model.

### 4.2. Structural Model and Main Effects

The structural model explained 44.9% of the variance in adoption intention (R^2^ = 0.449, adjusted R^2^ = 0.428), indicating moderate explanatory power. The Stone–Geisser Q^2^ value of 0.361 confirmed satisfactory predictive relevance. As shown in Figure 1, the path coefficients for all hypothesized and control paths are annotated on the research model, with solid lines denoting significant relationships and dashed lines denoting non-significant ones.

Hypothesis 1 was supported: privacy concerns exerted a significant negative effect on adoption intention (β = −0.281, SE = 0.052, t = 5.404, *p* < 0.001, f^2^ = 0.107), representing a small-to-medium effect size. Hypothesis 2 was also supported: perceived safety benefits had the strongest direct effect among all predictors (β = 0.358, SE = 0.049, t = 7.306, *p* < 0.001, f^2^ = 0.176), constituting a medium effect size. In absolute terms, the effect of perceived safety benefits exceeded that of privacy concerns. Among the control variables, technology trust (β = 0.152, *p* = 0.001), smart device experience (β = 0.121, *p* = 0.007), and perceived vulnerability (β = 0.091, *p* = 0.048) reached statistical significance. Children’s support also showed a significant direct path to adoption intention (β = 0.112, *p* = 0.015). The remaining demographic controls (age, gender, education, income, and duration of living alone) were not statistically significant at the 0.05 level. Health status likewise did not exhibit a significant direct effect on adoption intention (β = −0.068, *p* = 0.157). Table 3 reports the complete set of structural model results.

### 4.3. Moderation Effects and Supplementary Analyses

Table 3 (Panel B) reports the results of the two hypothesized moderation effects. Both interactions were significant, supporting Hypotheses 3 and 4.

**Figure 2:**
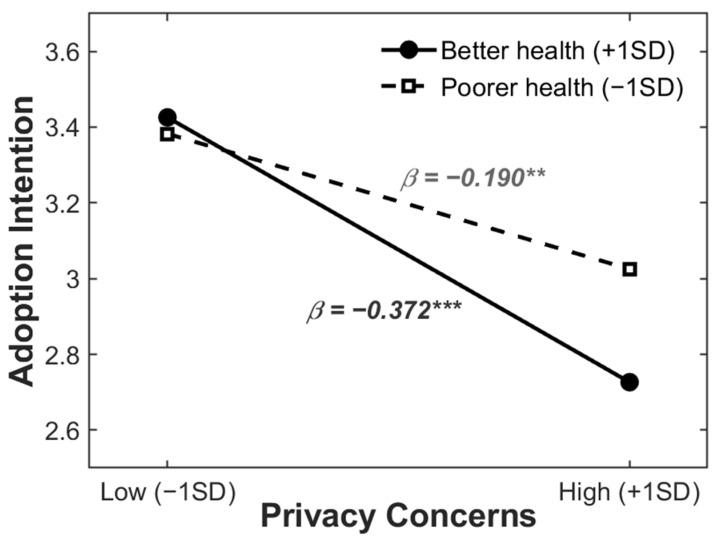
Simple Slope Plot: Moderating Effect of Health Status on the Relationship between Privacy Concerns and Adoption Intention. ** *p* < 0.01, *** *p* < 0.001.

Hypothesis 3 posited that health status moderates the negative effect of privacy concerns on adoption intention such that poorer health weakens this negative relationship. The interaction term was significant (β = −0.091, SE = 0.044, t = 2.068, *p* = 0.039, f^2^ = 0.027). Because health status was coded with higher values representing better health, the negative interaction coefficient indicates that as health deteriorates, the suppressive effect of privacy concerns on adoption intention diminishes. Simple slope analysis (Figure 2) confirmed this pattern: the slope of privacy concerns on adoption intention was steepest among participants with better health (β = −0.372, t = 5.723, *p* < 0.001) and substantially flattened among those with poorer health (β = −0.190, t = 2.836, *p* = 0.005). That is, older adults in poorer health were less deterred by privacy concerns when evaluating smart home adoption.

**Figure 3:**
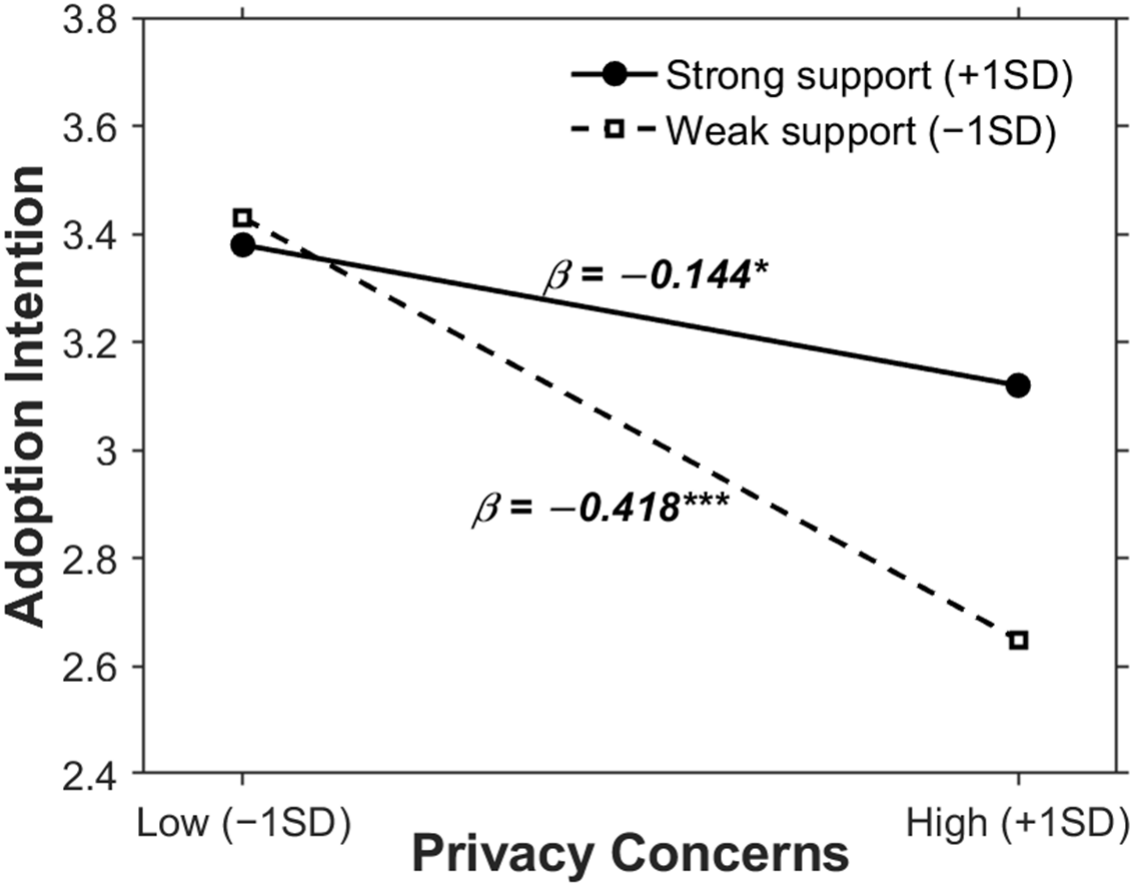
Simple Slope Plot: Moderating Effect of Children’s Support on the Relationship between Privacy Concerns and Adoption Intention. * *p* < 0.05, *** *p* < 0.001.

Hypothesis 4 proposed that children’s support moderates the same focal relationship, with stronger support attenuating the negative effect. The interaction term was significant and carried the largest moderation effect in the model (β = 0.137, SE = 0.043, t = 3.186, *p* = 0.001, f^2^ = 0.051). Simple slope analysis (Figure 3) revealed that at weak support (−1 SD), privacy concerns exerted a steep negative effect (β = −0.418, t = 6.239, *p* < 0.001), whereas at strong support (+1 SD), the same effect was reduced by roughly two-thirds (β = −0.144, t = 2.286, *p* = 0.022). The effect remained statistically significant even at high levels of support, suggesting that children’s involvement buffers rather than eliminates privacy-related resistance.

An importance–performance map analysis (IPMA) was conducted with adoption intention as the target construct. The results indicated that perceived safety benefits (importance = 0.341, performance = 69.5) and privacy concerns (importance = 0.263, performance = 63.0) occupied the high-importance zone, while technology trust (importance = 0.148, performance = 58.2) fell into a medium-importance yet low-performance quadrant, suggesting it may represent a particularly actionable lever for intervention.

Finally, thematic coding of the open-ended responses (Cohen’s κ = 0.81) yielded three salient themes. First, participants frequently expressed a desire for family-mediated privacy management, describing scenarios in which adult children would oversee data access on their behalf. Second, a recurring tension surfaced between valuing safety monitoring and feeling surveilled in one’s own home. Third, several respondents voiced a preference for simpler, less intrusive device configurations that focused on emergency functions rather than comprehensive behavioral tracking.

## 5. Discussion

### 5.1. Discussion of Findings

Both main-effect hypotheses were supported. The findings corroborate the dual-pathway structure of the privacy calculus [13] and extend it to older adults living alone, a population for whom the stakes on both sides are objectively higher. That safety benefits carried a larger effect size than privacy concerns aligns with the theoretical expectation that benefit perceptions outweigh cost perceptions when the need for protection is acute, a pattern also observed in the broader AIoT context [14]. The IPMA results added practical nuance: technology trust (importance = 0.148, performance = 58.2) fell into a medium-importance yet low-performance quadrant, marking it as the variable with the greatest room for improvement. Qualitative responses reinforced the calculus logic, with participants frequently describing a tension between valuing safety monitoring and feeling surveilled, echoing the ethical unease documented by Tian et al. [15]. However, the mean adoption intention (M = 3.14, SD = 0.94) remained only modestly above the scale midpoint, suggesting that factors beyond the rational calculus—unfamiliarity with the technology (82.8% had no prior exposure to smart home products), emotional resistance, or generalized distrust—impose additional friction that the framework does not fully capture.

Hypothesis 3 was supported: health status moderated the negative effect of privacy concerns on adoption intention (β = −0.091, *p* = 0.039). Simple slope analysis revealed that privacy concerns suppressed adoption intention far more steeply among participants in better health (β = −0.372) than among those in poorer health (β = −0.190). This pattern aligns with the observations of Wilkowska et al. [8], who found that older adults with more pressing health needs assigned less weight to privacy risks when evaluating ambient health systems. Interpreted through protection motivation theory [22], the simple slope difference (a drop from β = −0.372 to β = −0.190) suggests that participants in poorer health weighted safety gains more heavily in their adoption calculus, effectively discounting privacy costs when the perceived threat to personal safety was high. Bertolazzi et al. [37] documented a parallel pattern in an integrative review of chronic disease populations, reporting that older adults managing multiple conditions exhibited higher receptivity to health technologies precisely because the salience of health management overrode privacy reservations. The effect size was small (f^2^ = 0.027), but this figure should be read against a feature of the sample rather than taken as a sign that the mechanism itself is weak. With 85.6% of participants reporting at least one chronic disease and more than half reporting two or more (Table 1), responses on the health status scale clustered toward the poorer-health end (M = 2.83 on a five-point scale), leaving relatively little variation at the healthier end to work with. An interaction can only be estimated across the range of moderator values that actually appear in the data, and when that range is narrow, the size of the interaction term is pulled downward. That a significant interaction still emerged under these conditions suggests the present f^2^ sits closer to a floor than to a true estimate of how strongly health moderates the privacy effect. A sample covering a wider health gradient, including healthier community-dwelling older adults, would probably show a stronger effect. An alternative interpretation warrants consideration: chronic illness burden may reduce cognitive bandwidth for deliberative decision-making, meaning that older adults in poorer health may have been less deterred by privacy concerns not because they rationally discounted privacy but because they had fewer resources to engage with it. The data cannot adjudicate between these mechanisms, and this ambiguity carries ethical implications for interface design, as discussed below.

Hypothesis 4 received the strongest empirical support among the two moderation effects (β = 0.137, *p* = 0.001, f^2^ = 0.051). At weak children’s support, privacy concerns exerted a steep negative effect on adoption intention (β = −0.418), whereas at strong support the same effect was reduced by roughly two thirds (β = −0.144). This finding is consistent with prior evidence that intergenerational support facilitates smart home adoption [23,24], but extends it by identifying the pathway through which support operates: not merely as a direct promoter of adoption, but as a buffer that absorbs the deterrent force of privacy concerns. The buffering mechanism operates along two complementary channels: instrumentally, children help manage devices and oversee data access, transforming privacy from an uncontrolled risk into a delegated responsibility; emotionally, a child’s endorsement signals that risks are manageable. Qualitative responses confirmed this interpretation, with family-mediated privacy management emerging as the most salient theme. For solo-dwelling older adults, such support is delivered at a distance, constituting a social compensation mechanism that partially offsets the disadvantage of living alone [38]. Cui et al. [26] showed that different configurations of support produce distinct engagement patterns, suggesting that future research should disaggregate support by type and intensity. An important qualification is warranted: the negative effect of privacy concerns remained statistically significant even at strong support (*p* = 0.022), indicating that children’s involvement buffers rather than eliminates privacy-related resistance. This residual effect (β = −0.144, *p* = 0.022) is theoretically meaningful. It likely reflects privacy dimensions that fall outside the reach of family mediation, most notably concerns about third-party data misuse by technology companies or government agencies. While children can manage devices and oversee local data access, they cannot control how data are stored, processed, or shared once transmitted to cloud-based platforms. This suggests that children’s support addresses the relational layer of privacy concern (who in the older adult’s immediate circle sees their data) while leaving the institutional layer (what companies or authorities do with the data once it leaves the home) largely untouched. The distinction matters for design. Family co-management can be strengthened to cover the relational layer, but the institutional layer has to be handled separately—through clear data-handling agreements, enforceable limits on sharing with outside parties, and options that keep processing on the device rather than routing everything to the cloud. These points are developed in Section 5.2. Furthermore, children’s support was measured through self-report and may not correspond to actual support behaviors, a limitation that could attenuate the buffering effect at the behavioral adoption stage.

### 5.2. Theoretical and Practical Contributions

The study makes two theoretical contributions. First, it extends privacy calculus theory from direct-effect testing to the identification of moderation mechanisms. Prior applications of privacy calculus in the smart home and aging technology literatures have predominantly examined whether privacy concerns and perceived benefits predict adoption [7,13,14]. The present study shifts the analytical question from whether privacy concerns deter adoption to when they do so, revealing that their deterrent force is not fixed but varies systematically with the individual’s health profile and family support structure. This extension responds to a gap in the original framework of Culnan and Armstrong [12] and Dinev and Hart [13], which conceptualized the calculus as situationally variable but did not specify the moderating conditions that govern its operation. Second, the study contributes to an emerging research direction focused on the boundary conditions of negative psychological factors in technology adoption. Ezeudoka and Fan [9] demonstrated that anticipated regret moderates the deterrent effect of digital distrust on e-health resistance. The present findings extend this direction by identifying two additional boundary conditions, one rooted in physical vulnerability (health status) and one in social resources (children’s support), thereby broadening the evidence base for the proposition that negative adoption barriers operate conditionally rather than universally.

The empirical results carry four design and policy implications. At the product level, privacy controls should be differentiated by health profile. For healthier older adults, interfaces should offer layered options (e.g., enabling motion sensors while disabling cameras), ideally configured once at installation following an “install-time setup, daily hands-off” model. For those in poorer health, default configurations should emphasize a minimal viable monitoring profile—fall detection and emergency alerts enabled, behavioral tracking disabled unless opted in—accompanied by plain-language disclosure ensuring that simplified interfaces do not become a vehicle for uninformed consent. Relatedly, family co-management features—tiered data access permissions, delegated device management, and emergency notification chains—should be built into smart home products to institutionalize the support pattern identified in H4 [24]. Two boundary conditions apply: for older adults without available children, community workers could serve as proxy co-managers, leveraging institutional trust in grassroots governance structures; and to prevent power asymmetry, platforms should incorporate a “parent override” allowing the older adult to pause data sharing without disabling safety functions. At the community level, the IPMA singled out technology trust as a priority target, since it carried the lowest performance score among the significant predictors. What makes this actionable is that trust in this group tends to grow from concrete, observed experience rather than from abstract assurances and from sources that already belong to the older adult’s everyday social world. In urban China, the neighborhood committee (juweihui) and the senior university are exactly such sources, and their backing carries the credibility of familiar grassroots institutions. Trust-building is likely to work best when it is routed through them. A senior university can run hands-on courses where hesitant participants watch trusted peers operate a device and see for themselves what it does and does not record, and a neighborhood committee can vet and endorse specific products so that its screening stands in for a technical judgment older users often feel unable to make on their own. Disclosures about how data flow are better given in person at installation than buried in written agreements that many older users will not read [39]. At the policy level, smart home monitoring devices should be incorporated into China’s home aging-adaptation renovation subsidy program, which currently covers physical modifications but not digital safety equipment [40,41]. This would reduce the financial barrier for low-income participants (62.0% earned below 4000 RMB monthly) and signal governmental endorsement that addresses the trust deficit identified by the IPMA.

### 5.3. Limitations and Future Directions

Several limitations should be acknowledged. The cross-sectional, self-report design precludes causal inference and cannot capture how privacy attitudes evolve over time or translate into actual adoption behavior. Self-rated health may also diverge from clinical assessment. A further constraint is that the sample, though representative of the solo-dwelling older adults who most need monitoring, was heavily weighted toward people in poorer health; this narrowed the observed range of health status and limited the statistical sensitivity of the health moderation test, so its f^2^ is best read as a conservative estimate. Longitudinal designs linking survey responses to device logs and medical records, ideally in samples spanning a wider health gradient, would help address these issues.

Additionally, the Chinese context, shaped by Confucian filial norms, may amplify the moderating role of children’s support. Cross-cultural replications in more individualistic settings are needed to evaluate generalizability.

Moreover, the study treated “smart home technology” as a unified category, yet the privacy implications of a motion sensor differ markedly from those of an indoor camera. Participants in the open-ended responses explicitly expressed preferences for simpler, less intrusive device configurations focused on emergency functions rather than comprehensive behavioral tracking. Future research should disaggregate smart home technologies by how intrusive their data collection is and ask whether the privacy calculus shifts across device categories. Multi-group analysis (MGA) within PLS-SEM would suit this question well, since it allows a formal test of whether the structural paths—and the moderating effects in particular—differ significantly across configurations such as motion sensors, wearable alerts, and indoor cameras.

Finally, the community-based recruitment strategy excluded the most isolated older adults, whose privacy attitudes may differ substantially. Additionally, children’s support was measured solely through the older adults’ perceptions. Future research should employ door-to-door sampling to reach harder-to-access populations and adopt dyadic designs capturing both parent and child perspectives on support.

## 6. Conclusions

This study examined the conditions under which privacy concerns exert a stronger or weaker effect on smart home adoption intention among older adults living alone. Drawing on privacy calculus theory and surveying 326 solo-dwelling older adults across three Chinese cities, we tested two main-effect pathways and two moderation hypotheses using PLS-SEM.

The results confirmed that privacy concerns suppress adoption intention while perceived safety benefits promote it, with safety benefits exerting a larger effect. More critically, both moderation hypotheses were supported. Health status moderated the privacy barrier such that older adults in poorer health were less deterred by privacy concerns, consistent with the activation of protective motivation under conditions of physical vulnerability. Children’s support produced the strongest moderation effect in the model, reducing the deterrent force of privacy concerns by approximately two thirds when support was strong, a pattern attributable to both the instrumental delegation of data management and the emotional reassurance that family involvement provides. Together, these findings demonstrate that the privacy and safety trade-off is not fixed: it tilts in predictable ways depending on the older adult’s health profile and the intergenerational resources available to them.

Theoretically, the study advances privacy calculus from a direct-effect framework to one that incorporates moderation mechanisms, specifying when privacy concerns lose their deterrent power rather than merely confirming that they exist. Practically, the findings ground four actionable recommendations: health-differentiated privacy configurations with plain-language disclosure safeguards, built-in family co-management features with parental override mechanisms, trust-building through community-embedded experiential programs, and integration of smart home devices into China’s home aging-adaptation subsidy system. Ensuring that smart home technology reaches those who stand to benefit most—older adults who live alone, manage chronic conditions, and lack an immediate human safety net—is a necessary step toward universal access in an aging society.

## Figures and Tables

**Table 1:** Demographic Characteristics of the Sample (*n* = 326).

Characteristic	*n*	%
**Age (years), M = 71.4, SD = 6.2**		
60–64	52	15.9
65–69	78	23.9
70–74	89	27.3
75–79	68	20.9
≥80	39	12.0
**Gender**		
Male	124	38.0
Female	202	62.0
**Education**		
Primary school or below	87	26.7
Middle school	112	34.3
High school/vocational	83	25.5
College or above	44	13.5
**Monthly income (RMB)**		
<2000	68	20.9
2000–3999	134	41.1
4000–5999	82	25.2
≥6000	42	12.8
**Duration living alone (years)**		
<1	29	8.9
1–3	78	23.9
3–5	96	29.4
5–10	82	25.2
>10	41	12.6
**Number of chronic diseases**		
0	47	14.4
1	98	30.1
2	102	31.3
≥3	79	24.2
**Smart device experience**		
None	72	22.1
Basic smartphone only	198	60.7
Some smart home experience	56	17.2

**Table 2:** Measurement Model Results.

***Panel A: Factor Loadings***									
**Construct**				**Item**			**Loading**		
Privacy Concerns (PC)				PC1			0.821		
				PC2			0.873		
				PC3			0.848		
				PC4			0.758		
Perceived Safety Benefits (PSB)				PSB1			0.867		
				PSB2			0.883		
				PSB3			0.872		
				PSB4			0.845		
Adoption Intention (AI)				AI1			0.908		
				AI2			0.912		
				AI3			0.905		
Health Status (HS)				HS1			0.847		
				HS2			0.836		
				HS3			0.762		
Children’s Support (CS)				CS1			0.856		
				CS2			0.878		
				CS3			0.851		
				CS4			0.771		
***Panel B: Descriptive Statistics, Reliability, and Discriminant Validity***									
**Variable**	**M**	**SD**	**CR**	**AVE**	**1**	**2**	**3**	**4**	**5**
1. PC	3.52	0.89	0.896	0.684	**0.827**	0.284	0.467	0.196	0.312
2. PSB	3.78	0.82	0.924	0.753	−0.241	**0.868**	0.523	0.257	0.348
3. AI	3.14	0.94	0.934	0.825	−0.412	0.463	**0.908**	0.108	0.396
4. HS	2.83	0.91	0.852	0.659	0.158	−0.213	−0.087	**0.812**	0.185
5. CS	3.41	0.96	0.906	0.706	−0.267	0.298	0.347	−0.142	**0.840**

Note. (A) All factor loadings are significant at *p* < 0.001. (B) Diagonal (bold) = √AVE; below diagonal = Pearson correlations; above diagonal = HTMT. PC = Privacy Concerns; PSB = Perceived Safety Benefits; AI = Adoption Intention; HS = Health Status; CS = Children’s Support.

**Table 3:** Structural Model Results.

***Panel A: Direct Effects***						
**Path**	**β**	**SE**	**t**	***p***	**Result**	
PC → AI (H1)	−0.281	0.052	5.404	<0.001	Supported	
PSB → AI (H2)	0.358	0.049	7.306	<0.001	Supported	
HS → AI	−0.068	0.048	1.417	0.157	n.s.	
CS → AI	0.112	0.046	2.435	0.015	*	
Age → AI	−0.058	0.051	1.137	0.256	n.s.	
Gender → AI	0.027	0.048	0.563	0.574	n.s.	
Education → AI	0.084	0.044	1.909	0.057	n.s.	
Income → AI	0.069	0.049	1.408	0.159	n.s.	
Living alone → AI	−0.039	0.046	0.848	0.397	n.s.	
Tech experience → AI	0.121	0.045	2.689	0.007	**	
Tech trust → AI	0.152	0.047	3.234	0.001	**	
Perceived vulnerability → AI	0.091	0.046	1.978	0.048	*	
***Panel B: Moderation Effects***						
**Interaction**	**β**	**SE**	**t**	***p***	**f^2^**	**Result**
PC × HS → AI (H3)	−0.091	0.044	2.068	0.039	0.027	Supported
PC × CS → AI (H4)	0.137	0.043	3.186	0.001	0.051	Supported

Note. *n* = 326. Bootstrap samples = 5000. R^2^ = 0.449, Adjusted R^2^ = 0.428, Q^2^ = 0.361. * *p* < 0.05, ** *p* < 0.01. PC = Privacy Concerns; PSB = Perceived Safety Benefits; AI = Adoption Intention; HS = Health Status; CS = Children’s Support. n.s. = not significant.

## Data Availability

The data presented in this study are available upon request from the corresponding author. The data are not publicly available because they contain sensitive personal and health-related information from a vulnerable population of older adults living alone, and the informed consent obtained and the ethics approval granted for this study did not authorize public deposition of individual-level data. De-identified data may be made available to qualified researchers upon reasonable request and subject to a data-use agreement.
